# Undercarboxylated osteocalcin does not correlate with insulin resistance as assessed by euglycemic hyperinsulinemic clamp technique in patients with type 2 diabetes mellitus

**DOI:** 10.1186/1758-5996-4-53

**Published:** 2012-12-18

**Authors:** Katsuhito Mori, Masanori Emoto, Koka Motoyama, Eiko Lee, Shinsuke Yamada, Tomoaki Morioka, Yasuo Imanishi, Tetsuo Shoji, Masaaki Inaba

**Affiliations:** 1 Deaprtment of Metabolism, Endocrinology and Molecular Medicine, Osaka City University Graduate School of Medicine, 1-4-3, Asahi-machi, Abeno-ku, Osaka, 545-8585, Japan

**Keywords:** Osteocalcin, Undercarboxylated osteocalcin, Insulin resistance, Type 2 diabetes

## Abstract

**Background:**

Recent *in vitro* and *in vivo* studies have suggested a critical role of osteocalcin (OC), especially the undercarboxylated form (ucOC), in insulin secretion and insulin sensitivity. The objective of this study was to investigate the association between serum ucOC levels and insulin resistance in humans with type 2 diabetes mellitus.

**Findings:**

We measured serum ucOC levels in 129 patients with type 2 diabetes. Insulin resistance was assessed using the euglycemic hyperinsulinemic clamp technique. The insulin resistance indices used were the M value, which is the total body glucose disposal rate, and the M/I value, which is the M value adjusted for the steady state plasma insulin level. ucOC levels were not correlated with the M value (ρ = −0.013, p = 0.886) or the M/I value (ρ = 0.001, p = 0.995).

**Conclusions:**

We found no association between ucOC levels and insulin resistance in patients with type 2 diabetes mellitus.

## Background

Emerging evidence has shed light on the role of bone as an endocrine organ that regulates energy metabolism [1,2]. Among its intermediate factors, bone-derived osteocalcin (OC) has attracted much attention. Recent vigorous studies have demonstrated that OC regulates both insulin secretion and insulin sensitivity [3-5]. Clinically, serum OC level is utilized as a bone formation marker, because OC is synthesized by osteoblast [1]. Therefore, vitamin-K dependent gamma-carboxylated OC, which binds to hydroxyapatite in bone [1], has been considered to be active in bone metabolism. Intriguingly, *in vitro* and *in vivo* studies have clearly demonstrated that undercarboxylated form of OC (ucOC) is involved in glucose metabolism in rodents [3-5]. Most studies in humans have suggested that higher OC levels are associated with better metabolic profiles as assessed by cretain parameters, such as fasting plasma glucose (FPG), hemoglobin A1c (HbA1c), the homeostasis model assessment (HOMA)–β, and the HOMA of insulin resistance (HOMA-IR) [1,2]. However, data that examines the correlation between ucOC kevels and glucose metabolism is limited. Kanazawa et al. reported that ucOC levels were negatively associated with FPG and HbA1c [6]. Focusing on insulin resistance, one report suggested that total OC and carboxylated OC levels, and not ucOC levels, were associated with the HOMA-IR [7]. In contrast, Iki et al. found an inverse correlation between ucOC levels and the HOMA-IR [8]. These contradictory findings may have arisen from methodological limitations in their approaches for evaluating insulin resistance and the particular cohorts examined, which were basically designated for osteoporosis research.

Therefore, we examined whether serum ucOC levels are associated with insulin resistance in patients with type 2 diabetes using M and M/I values, which are gold standard indices for measuring insulin resistance, using the euglycemic hyperinsulinemic clamp technique.

## Methods

A total of 129 subjects were selected from participants admitted our diabetes center at Osaka City University Hospital. Patients with serum creatinine levels >1.2 mg/dL (106 μmol/L) and other active medical diseases were excluded. Of the 129 patients selected, 65 were taking the following medications: insulin (n = 15), sulfonylureas (n = 29), α-glucosidase inhibitors (n = 5), biguanides (n = 1), insulin secretagogues (n = 3), thiazolidinedione (n= 2), and combination therapies of these drugs (n = 10). The study protocol was approved by the local ethics committee, and informed consent was obtained from all participants prior to study initiation.

HbA1c (%) levels were estimated as National Glycohemoglobin Standardization Program equivalent values (%) and were calculated by the formula HbA1c (%) = HbA1c (JDS;%) + 0.4%, considering the relative expression of HbA1c (%) as measured by standard laboratory methods and previous Japanese standard materials [9]. ucOC levels were measured by electrochemiluminescence immunoassay (Picolumi ucOC, Sanko Junyaku Co. Ltd., Tokyo, Japan) [6][8]. Insulin resistance was assessed by the euglycemic hyperinsulinemic clamp using an STG 22 artificial pancreas model (Nikkiso Co., Tokyo), as described previously [10,11]. The total body glucose disposal rate was determined as the mean of the glucose infusion rate (M) during the last 30 minutes of the clamp. The insulin resistance index (M/I value) was calculated by dividing the mean M by the steady state plasma insulin level during the last 30 minutes of the clamp and multiplying by 100. All values are reported as mean ± the standard deviation (SD unless otherwise indicated. Because the ucOC, M, and M/I values were not normally distributed, we used Spearman rank correlation test to study the association between the ucOC, M, and M/I values. The Mann–Whitney *U* test was used to compare ucOC levels between male and female subjects. *P* values of <0.05 were considered statistically significant.

## Results

**Figure 1:**
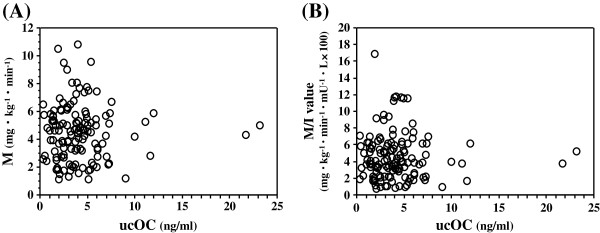
**The association between serum ucOC levels and M (A) and M/I values (B).** There was no significant association of serum ucOC levels and M (ρ = −0.013, p = 0.886) and M/I values (ρ = 0.001, p = 0.995).

Clinical characteristics of the subjects are summarized in Table 1. Median ucOC levels were 3.7 ng/mL (interquartile range [IQ], 2.2-5.1) and ranged from 0.4 to 23.1 ng/mL. The median M values and M/I values were 4.6 (IQ, 2.8-5.7; range, 1.1 to 10.8) mg·kg^-1^·min^-1^ and 4.0 (IQ, 2.4-6.1; range, 0.7 to 16.9) mg·kg^-1^·min^-1^·mU^-1^·L×100, respectively. ucOC levels were not correlated with either the M value (ρ = −0.013, p = 0.886) (Figure 1A) or the M/I value (ρ = 0.001, p = 0.995) (Figure 1B). Because ucOC levels were significantly lower in males than in females (males, 3.4[IQ, 2.0-4.6]; females, 4.2[IQ, 2.9-5.9]; p = 0.018), we divided all patients into two groups by sex and examined the association between ucOC levels and M values and M/I values. We found that ucOC levels were not correlated with M values in both males (ρ = −0.077, p = 0.500) and females (ρ = 0.065, p = 0.650). There was also no correlation between ucOC levels and M/I values in both males (ρ = −0.052, p = 0.648) and females (ρ = 0.068, p = 0.632).

**Table 1:** Clinical characteristics and insulin resistance parameters measured with the hyperinsulinemic euglycemic clamp technique in subjects with type 2 diabetes

Age (years)	54.9 ± 12.3
Sex (Ma/Fe)	79/50
Duration of Diabetes (years)	6.0 (2.0–12.0)
BMI (kg/m2)	25.2 ± 2.8
SBP (mmHg)	127 ± 18
FPG (mg/dL)	134 (117–160)
HbA1c (%)	9.0 ± 2.2
TC (mg/dL)	196 ± 32
HDL (mg/dL)	43 (35–51)
TG (mg/dL)	122 (92–159)
sCre (mg/dl)	0.71 ± 0.17
M (mg·kg^-1^·min^-1^)	4.6 (2.8–5.7)
SSPI (μU/mL)	104 (86–127)
M/I value (mg·kg^-1^·min^-1^·mU^-1^·L×100)	4.0 (2.4–6.1)

All values are expressed as mean ± SD, n, or median (interquartile range). *BMI*: body mass index, *SBP*: systolic blood pressure, *FPG*: fasting plasma glucose, *TC*: total cholesterol, *HDL*: high density lipoprotein, *TG*: triglyceride, *sCre*: serum creatinine, *M*: glucose infusion rate, *SSPI*: steady state plasma insulin, *M/I* value: defined by dividing M by SSPI and multiplying by 100.

## Discussion

This is the first study, as per our knowledge, to examine the association between ucOC levels and insulin resistance using the euglycemic clamp technique, which is the gold standard method for evaluating insulin resistance in humans. We found no significant correlation between ucOC levels and insulin resistance in patients with type 2 diabetes. These findings suggest that it is premature to conclude that ucOC plays a role in glucose metabolism in humans.

The HOMA-IR is easily calculated from fasting insulin and glucose levels and is commonly used for the evaluation of insulin resistance in clinical practice. However, some attention needs to paid to the interpretation of these values as indices of insulin resistance because they mainly depend on the balance between hepatic glucose output and insulin secretion, which is maintained by a feedback loop between the liver and pancreatic β-cells [12]. In particular, the HOMA-IR may not be a good tool for this purpose because ucOC levels appear to affect both insulin secretion and insulin sensitivity concurrently. On the other hand, M/I values predominantly represent insulin resistance in insulin-sensitive organs, such as skeletal muscle and adipose tissue, but not in the liver. This is because insulin reaches approximate level of 100 μU/mL under euglycemic hyperinsulinemic clamp procedures; therefore, they can almost completely suppress hepatic glucose output [10,11]. Indeed, the average plasma insulin level achieved in our study was 104 μU/mL (Table 1). Therefore, M/I values are accurate and quantitative in the assessment of insulin resistance, primarily in skeletal muscle, and are independent of insulin secretion capacity in humans. However, this procedure is time-consuming, costly, and complicated.

Administration of recombinant ucOC into mice clearly enhanced both insulin secretion and sensitivity [3-5]. However, its impact on glucose metabolism in humans remains unclear. Although we targeted subjects with apparent type 2 diabetes, this may confound the interaction between ucOC levels and insulin resistance. For example, diabetes has been linked to dysregulated bone metabolism [2]. Another possible confounding factor may be treatment with antidiabetic drugs. Insulin regulates the function of osteoblasts and the production of OC/ucOC [2]. Furthermore, a recent longitudinal study in elderly men, including those with diabetes, showed that the increase in ucOC levels was associated with improvements in the HOMA-IR, and this association was limited to subjects who were not treated with antidiabetic drugs [13]. Therefore, aberrant glucose metabolism, drug interventions or both may make it more difficult to interrupt the correlation between ucOC levels and insulin resistance. In this regard, Iki et al. clearly showed a significant inverse association between ucOC levels and the HOMA-IR in community-based population without apparent health problems [8]. If the effect of ucOC on glucose metabolism is not comparable to that of strong influential factors such as adiponectin, which is a key player in energy metabolism [14], cohort selection may be critical in confirming the association between ucOC levels and insulin secretion and sensitivity.

The present study had several limitations. First was the cross-sectional study design and the relatively-small sample size. Second, there was no information regarding medications for metabolic bone diseases, including vitamin K, which up-regulates gamma-carboxylation of OC. Third, we did not measure total OC levels. However, the ucOC/OC ratio may be a more relevant measure. Finally, although we hypothesized that ucOC could affect insulin sensitivity via adiponectin, serum adiponctin levels were not determined.

In conclusion, we found no association between ucOC levels and insulin resistance in patients with type 2 diabetes as per the euglycemic hyperinsulinemic clamp technique. Further studies are required to confirm the impact of ucOC, OC, and ucOC/OC ratio on insulin resistance in humans.

## Abbreviations

OC: Osteocalcin; ucOC: Undercarboxylated form; HOMA-β: Homeostasis model assessment–β; HOMA-IR: Homeostasis model assessment of insulin resistance; BMI: Body mass index; SBP: Systolic blood pressure; FPG: Fasting plasma glucose; TC: Total cholesterol; HDL: High density lipoprotein; TG: Triglyceride; sCre: Serum creatinine.

## Competing interest

The authors declare that they have no competing interests.

## Authors’ contributions

Mori conceived of the research hypothesis and performed the statistical analyses, and wrote the manuscript. Emoto assisted in conception of the research hypothesis and in writing the manuscript. Motoyama and Lee collected the data. Yamada, Morioka, Imanishi, Shoji and Inaba reviewed and edited the manuscript. All authors read and approved the final manuscript.
